# High-speed axially swept light sheet microscopy using a linear MEMS phased array for isotropic resolution

**DOI:** 10.1117/1.JBO.25.10.106504

**Published:** 2020-10-23

**Authors:** Joseph Landry, Stephen Hamann, Olav Solgaard

**Affiliations:** Stanford University, Edward L. Ginzton Laboratory, Stanford, California, United States

**Keywords:** light sheet microscopy, phased array, axial scan, cylindrical lens

## Abstract

**Significance:** Axially swept light sheet microscopy is used for deconvolution-free, high-resolution 3D imaging, but usually the axial scan mechanism reduces the top imaging speed. Phased arrays (PAs) for axial scanning enable both high resolution and high speed.

**Aim:** A high-speed PA with an update rate faster than the camera row read time is used to track the rolling shutter at camera-limited rates.

**Approach:** The point spread function is evaluated to ensure sub-micron isotropic resolution, and the technique is demonstrated on a live *Drosophila* embryo.

**Results:** Isotropic resolution is shown down to 720±55  nm in all three spatial dimensions. With an update rate of 2.85  μs, the PA tracks the camera sensor rolling shutter at camera-limited rates. Features in the *Drosophila* embryo are resolved clearly compared with the equivalent static light sheet case. The random-access nature of the PA enables a camera sensor readout in the same direction for each frame to maintain even temporal sampling in image sequences with no speed loss.

**Conclusions:** Use of PAs is compatible with axially swept light sheet microscopy and offers significant improvements in speed.

## 1 Introduction

Light sheet fluorescence microscopy (LSFM) has become a valuable 3D imaging tool for biological scientists due to its high throughput and favorable photobleaching rates.^1^^–^^4^ These two properties make LSFM a favorable alternative to confocal and two-photon microscopy for long-term 3D imaging of transparent and semi-transparent specimens, such as cleared tissue or developing embryos.^5^^,^^6^ Although LSFM excels at volumetric imaging, traditional Gaussian beam-based light sheet systems commonly have poor axial resolution due to the trade-off between light sheet uniformity and thickness due to the nature of the Gaussian beam.

Many techniques address this trade-off by constructing the light sheet from non-Gaussian beam profiles. Scanned Bessel beams^7^^–^^9^ and optical lattices^10^ produce uniform sheets over the sample field of view (FOV) with narrow central lobes. However, these techniques result in side lobes, producing significant out-of-focus blurring that requires 3D deconvolution to recover the resolution and prolongs recovery of the final image. Other techniques capture multiple image volumes from different views.^4^^,^^11^^,^^12^ The views are used in post-processing to reconstruct the final image volume with the most detailed information from each view. While effective, these methods can reduce imaging speed, require significantly more data storage, and do not provide real-time imaging.

Deconvolution-free sub-micron isotropic resolution can be achieved using axially swept light sheet fluorescence microscopy (ASLM).^13^ In ASLM, a narrow, uniform light sheet is produced by scanning a tightly focused Gaussian beam along the illumination optical axis and suppressing the out-of-focus regions. The original ASLM methods fused the in-focus regions of a series of images with different light sheet foci to produce an overall in-focus image.^14^^–^^16^ More recent implementations have synchronized a continuously moving beam to the rolling shutter of an sCMOS camera, allowing the shutter to reject the out-of-focus light. These techniques have been demonstrated using piezo reference mirrors,^13^ electrically tunable lenses,^17^ and voice-coil stages.^18^ All of these methods require large mechanical movements with low speeds that cannot match the maximum speed of the camera. Further, these techniques do not scale well into faster frame rates, such as those available at reduced camera areas of interest, as each pass of the scanning element imposes significant downtime. One recent demonstration uses a 2D ferroelectric spatial light modulator, but the 1-kHz update rate is not fast enough to update at the same rate as the rolling shutter.^19^

In this paper, we demonstrate a camera-limited axially swept light sheet microscope with isotropic resolution down to 720 nm using a linear phased array (PA) with an update rate of 350 kHz. The maximum update rate of this device (2.85  μs) is faster than the update rate of our camera rolling shutter (9.6  μs), which ensures the camera frame rate is matched at full-frame and any smaller area of interest. As a random access device, the scan can be performed in the same direction for each frame acquisition with no speed penalty, which is important for maintaining even temporal sampling during time lapses.

## 2 Background

When using Gaussian beams to form the light sheet in LSFM, the beam width is ordinarily chosen such that the confocal range $x_{c}$, defined as twice the Rayleigh range, is at least equal to the FOV in the object plane. To create a uniform sheet at wavelength λ, the beam waist $w_{0}$ must be chosen such that

$$w_{0}\geq \sqrt{\frac{x_{c}\lambda }{2\pi }}.$$ (1)

**Fig. 1:**
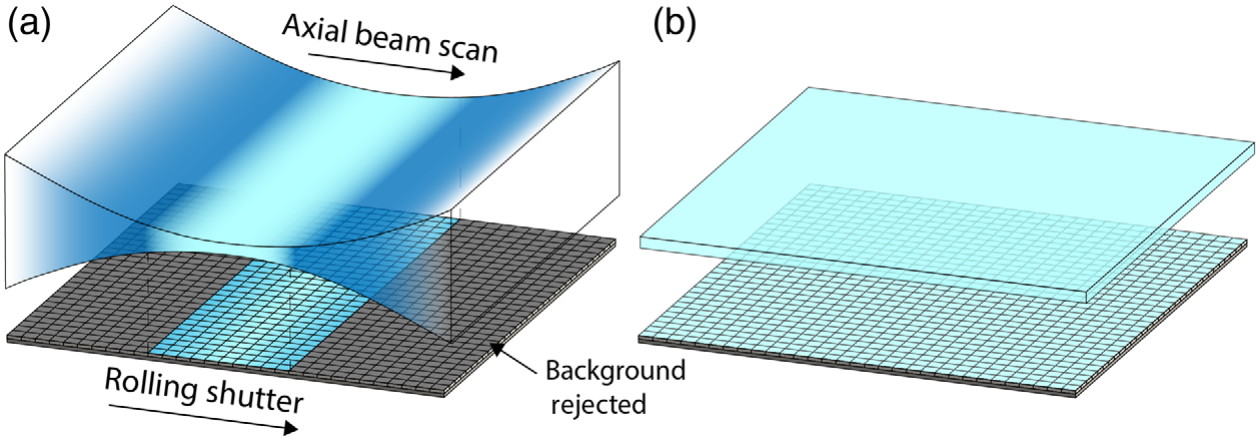
Rolling shutter synchronization concept showing the light sheet scan in the sample plan and camera sensor at the image plane. (a) A point in time during the camera acquisition in which a 10-pixel rolling shutter is synchronized to the axial scan of a Gaussian beam tightly focused in 1D. (b) The narrow light sheet resulting from only imaging the tightly focused region and rejecting out-of-focus light.

This resulting beam width is usually several times larger than the width required for the axial resolution to approach the lateral resolution. With ASLM, the light is focused tightly, resulting in a confocal range that is uniform over a small fraction of the FOV at any given time. By axially scanning the focus of this beam at the center of the camera’s rolling shutter, as illustrated in Fig. 1, an effective light sheet that is both uniform and narrow is produced. The width of the shutter is set to $Mx_{c}$, where M is the collection path magnification, to reject the out-of-focus light.

**Fig. 2:**
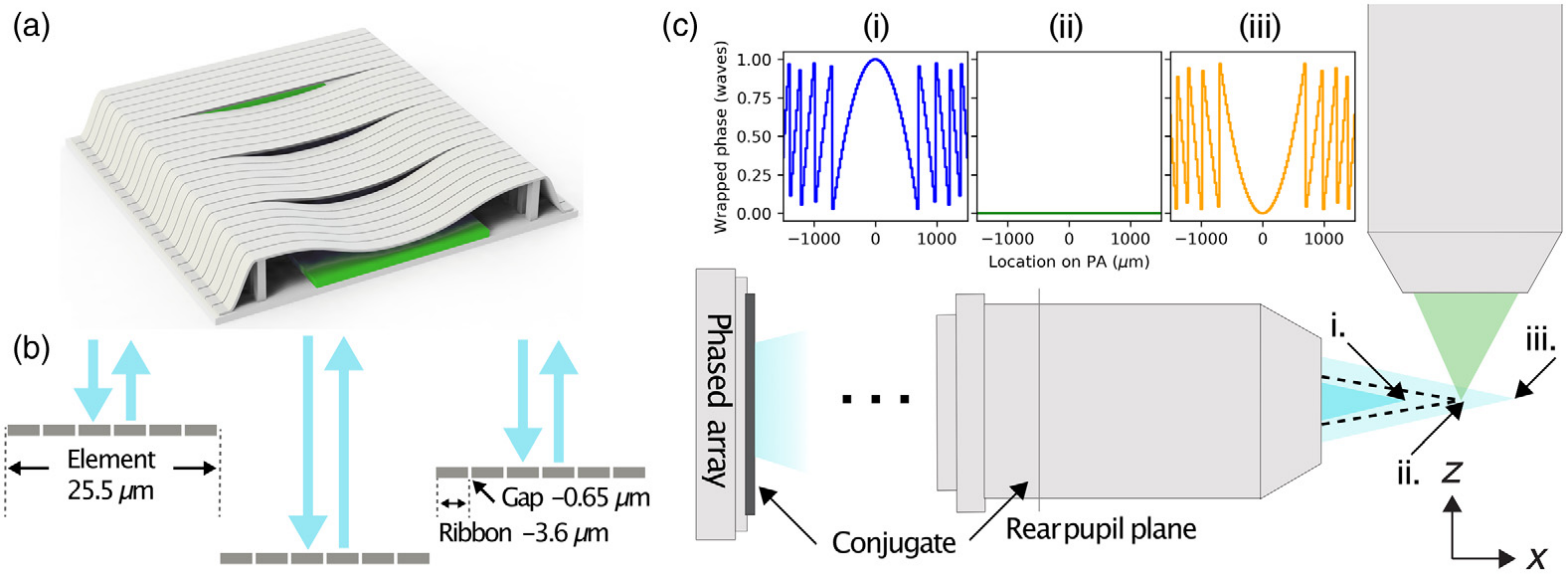
Cylindrical lensing using a PA. (a) Isometric render showing four of the 1088 elements, three of which are actively deflecting toward the common electrode. (b) Cross section along the long axis of the device showing the six-ribbon substructure of each element, all of which actuate together. At normal incidence, the phased modulation provided by an element is given by $\frac{2d}{\lambda }$ waves, where d is the element displacement. (c) Axial scanning with the PA conjugate to the rear pupil of the IO. The dashed line shows the nominal focus with the PA in the quiescent state. (i)–(iii) The resulting light sheet focus locations under (i) positive power, (ii) zero power (bias point), and (iii) negative power cylindrical lensing.

The device used for axial beam scanning is a reflective, linear MEMS PA [Figs. 2(a) and 2(b)]. The array consists of 1088 electrostatically driven elements that are 25.5  μm wide and are composed of six aluminum-coated silicon nitride (${\mathrm{Si}}_{3}N_{4}$) ribbons. The ribbons are 3.6  μm wide with a 0.65-μm gap between them. Each element is individually controllable and capable of deflecting up to 250 nm with 10-bit precision, providing fine phase control from 0 to 2π round-trip phase shift for wavelengths as long as 500 nm. The PA is random access, meaning that the entire state of the PA is switchable in the time it takes to change a single element: 2.85  μs or 350 kHz. The speed of the PA is electronically limited and can, in principle, operate in the MHz.^20^

Axial scanning with this PA is performed by placing it conjugate to the rear pupil plane of the illumination objective (IO) and displaying phase patterns with different effective lens powers in series. Displaying positive or negative phase curvatures results in translation of the line beam focus about a bias point [Fig. 2(c)]. The phase profile $\phi_{c}$ for a desired focal length f is given by

$$\phi_{c}(p,f)=\{\begin{array}{cc} \frac{2\pi }{\lambda }(f-\sqrt{f^{2}+p^{2}}) & f>0\\ \frac{2\pi }{\lambda }(f+\sqrt{f^{2}+p^{2}}) & f<0 \end{array},$$ (2)

where p is the transverse element position on the PA relative to the optical axis. Treating the PA as an adjustable thin lens in the paraxial regime and propagating the reflected beam using ray matrices, the change in focal position about the bias point is given by

$$\Delta z=-n\frac{f_{IO}^{2}}{f_{IO}+f_{PA}-u},$$ (3)

where $f_{IO}$ and $f_{PA}$ are the focal lengths of the IO and PA, respectively, u is the distance between them, and n is the refractive index of the objective immersion medium. With a well-aligned system in which the PA is exactly conjugate to the IO rear pupil plane, u becomes equal to $f_{IO}$ and Eq. (3) becomes linear with PA lens power $-nf_{IO}^{2}P_{PA}$. To first order, the numerical aperture (NA) of the scanned illumination beam in this configuration is independent of PA power. As long as the system aperture is large enough to accommodate the change in beam size caused by the PA, the beam waist of the scanned line beam remains unchanged along its axis.

The discrete states of the PA are perfectly suited for synchronization with the camera rolling shutter as the rolling shutter itself advances in discrete jumps at fixed intervals. For optimal spatial overlap, each change in shutter position should be followed by an update of the PA to position the beam at the new center of the shutter. The fastest line rate of our sCMOS camera is 9.6  μs, which sets the maximum rate at which the shutter advances. Thus, the PA, which updates as fast as 2.85  μs, can readily keep up. Because the PA can jump between any two arbitrary states, there is no duty cycle caused by a fixed reset period, nor is it required to alternate the scan directions with each frame to preserve speed. Importantly, this random access nature also means that there is no distinction between operating the camera at 50 Hz in full-frame or hundreds of Hz at smaller camera sensor regions of interest; all are camera-limited.

Due to the discrete, flat-phase elements that make up the array, it is not possible to produce phase curvatures that track Eq. (2) exactly. The greater the slope of $\phi_{c}(p,f)$ is, the greater the phase deviation over each element will be. The total phase error aggregates the error from all of the actively illuminated elements in the array. Thus, larger beams, which cover elements more distant from the optical axis and thus have high phase gradients, result in higher wavefront error. Short focal lengths, which increase the phase gradient for all elements, will also increase the wavefront error. We previously reported the axial scanning limits using this linear PA for cylindrical lensing.^21^ Given a beam collimated along the long axis of the PA, the array can produce a cylindrically converging or diverging reflected beam with a maximum diffraction-limited NA of 0.008, which corresponds to a wavefront RMS error of 0.07 waves at 488 nm. After this NA, the beam quality deteriorates and light efficiency into the scanned focus drops significantly as light is redirected into higher orders. Depending on the applied NA, the PA has an overall efficiency of 50% to 70%. Changes to the PA architecture that decrease the element size enable the use of higher NA at higher efficiencies.

## 3 Experimental Setup

**Fig. 3:**
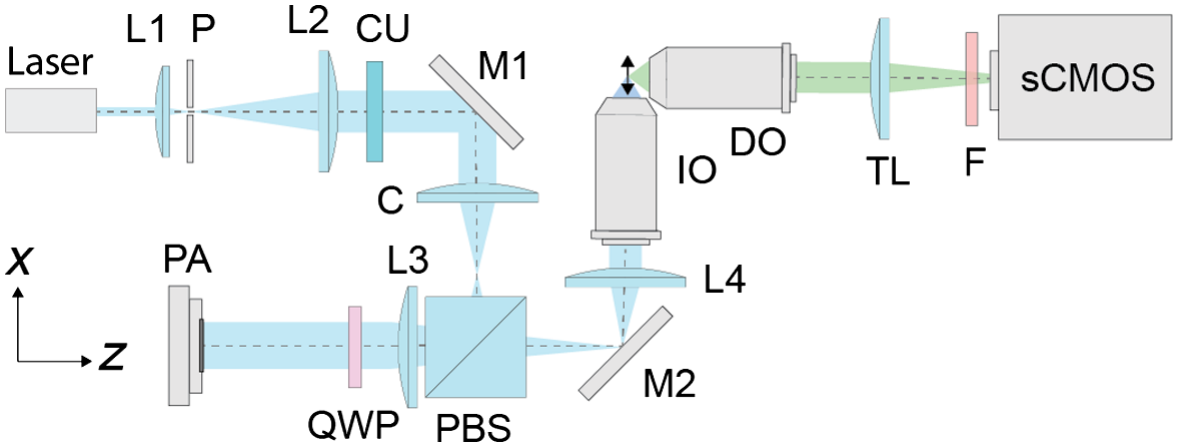
System overview. L1 to L4, achromatic doublet lenses; P, pinhole; CU, cleanup filter; M1 to M2, mirrors; C, cylindrical lens; PBS, polarizing beam splitter; QWP, quarter wave plate; PA, phased array; IO, illumination objective; DO, detection objective; TL, tube lens; and F, long-pass filter.

The full setup is shown in Fig. 3. A 488-nm laser (TOPTICA iBEAM smart, 0.41 mm $1/e^{2}$ radius, vertical linear polarization) is expanded 10.8× by L1 (7.4 mm) and L2 (80 mm) and spatially filtered by a 12.5-μm pinhole P (Edmund Optics, 38-539). The beam passes through cleanup filter CF (Semrock, FF01-488/10-25) and is reflected by a mirror toward cylindrical lens C (100 mm), which focuses the beam into a vertical line. A polarizing beamsplitter (PBS) reflects the vertically polarized beam, after which it is focused by L3 (100 mm) and is circularly polarized by a quarter wave plate (QWP). The beam is then incident on the horizontally oriented PA with width 4.5 mm $1/e^{2}$ radius along the length of the PA. After reflection, the phase-modulated beam passes through the QWP and L3 again, now passing through the PBS with horizontal polarization. After reflecting off mirror M2 and passing through L4 (80 mm), the beam, with a collimated radius dimension of 3.6 $1/e^{2}$, enters the IO (Olympus UMPlanFL 20×0.5  NA), resulting in an illumination NA of 0.37 $1/e^{2}$. Both the IO and the detection objective (DO, Olympus UMPlanFL 20×0.5  NA) water immersion objectives are fixed in an aluminum chamber that holds the specimen. Fluorescence collected by the DO passes through tube lens TL (Thorlabs TTL180-A), long-pass filter F (Semrock BLP01-488R-25) and forms an image on the sCMOS camera (Zyla 4.2 PLUS), with its rolling shutter oriented vertically.

Timing synchronization between the phase modulator and camera is handled by a microcontroller (Teensy 3.6), which acts as a master to the camera and PA. The PA controller memory is loaded with a predefined sequence of voltage values prior to image acquisition, which depend on the camera area-of-interest. Each predefined voltage pattern corresponds to a specific focal length of the PA. Upon acquisition start, the TTL input to the PA triggers this display update sequence. The microcontroller provides simultaneous triggers to the frame input of the camera and display sequence of the PA, initiating a free-run of the PA states and camera rows, which are set to change at the same rate. Each time the rolling shutter advances, the PA moves to the next voltage pattern in the sequence, changing the focal length, and thus the position, of the line beam to match the new location of the rolling shutter. The maximum frame rate at the full area of interest is 50 frames per second.

Spatial synchronization requires calibration of the PA sequence to match the camera’s known shutter position sequence for a given area of interest. This is performed by choosing three different lens powers on the PA, recording the row on the camera corresponding to the beam waist, and fitting to Eq. (3) to derive the required lens power to position the beam to any specific camera row.

## 4 Results

**Fig. 4:**
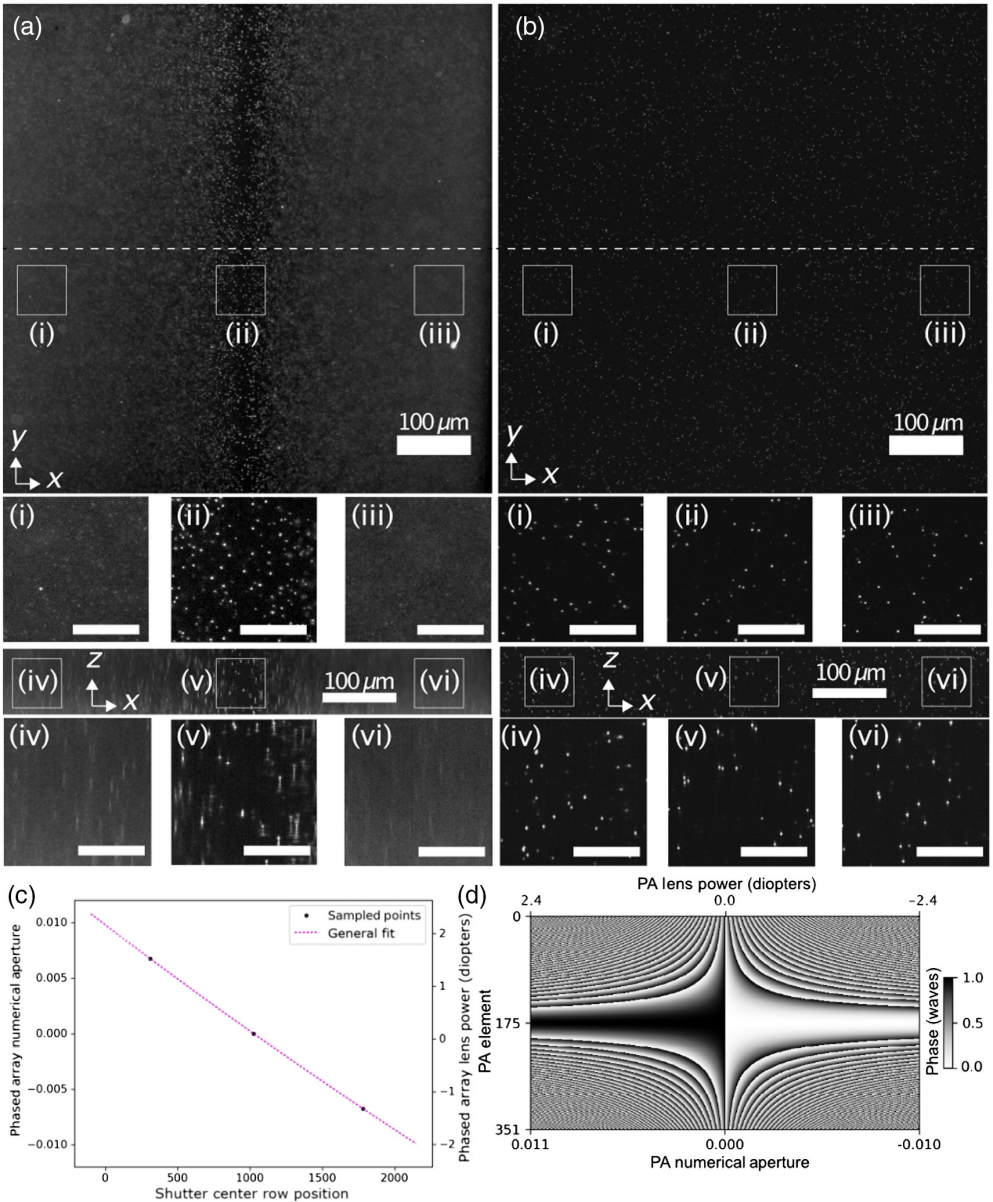
Spatial synchronization of the rolling shutter and axially scanned beam using 200-nm yellow-green fluorescent beads. Images of the beads show both lateral (XY) and axial (XZ) views, with (i)–(vi) showing close-ups of the respective regions with 30  μm scale bars. (a) The nominal focus of the light sheet with the PA off. (b) Image after synchronization of rolling shutter and beam focus. (c) Fitting focal location on camera to PA effective lens power. (d) The sequence of approximately 2048 lookup tables generated by the fit that track the rolling shutter as it advances across the shutter. Each column is a single state of the PA, which updates at the same rate as the camera shutter.

**Fig. 5:**
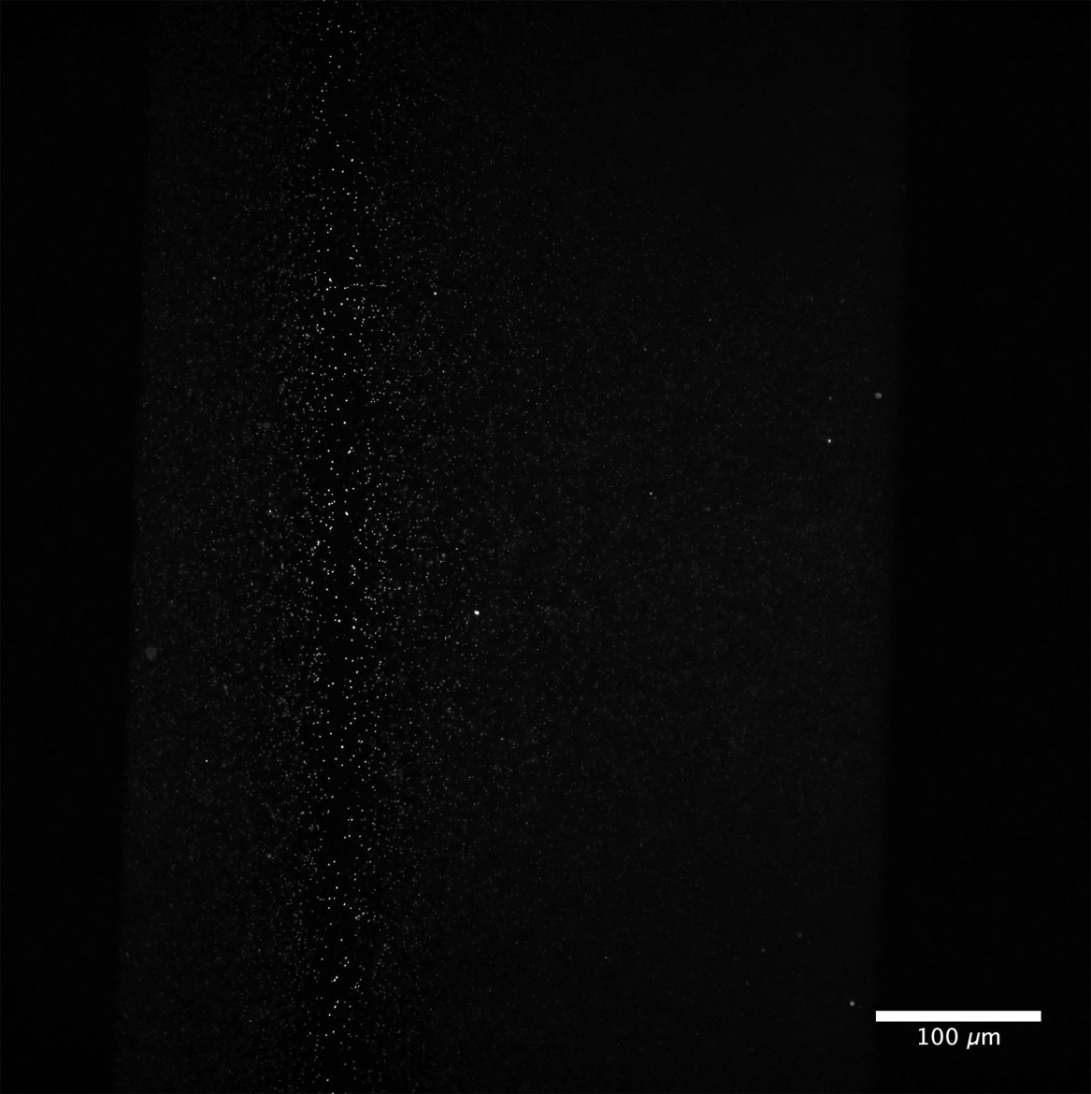
Light sheet axial scan across the FOV filled with 200-nm fluorescent beads. For this scanning demonstration, the PA updates once per camera frame (Video 1, MOV, 6.5 MB [URL: https://doi.org/10.1117/1.JBO.25.10.106504.1]).

An agarose suspension of yellow-green 200-nm fluorescent beads (ThermoFisher F8811) was prepared to evaluate the spatial synchronization of the beam with the rolling shutter of the camera. The image of the beads with the PA in the quiescent (mirror-like) state is shown in Fig. 4(a), in which the beam converges to form a tight focus in the image center. Synchronization of this focus to the rolling shutter resulted in the rejection of out-of-focus light and sharp beads across the FOV [Fig. 4(b)]. Synchronization was achieved by fitting Eq. (3) using the waist positions recorded for lens powers of −1.5, 0, and 1.5 diopters with pixel rows 310, 1025, and 1780. This calibration maps each row location to the necessary PA lens power required to focus at that location [Fig. 4(c)]. The known shutter positions are used to populate the corresponding lens power display sequence on the PA to track the rolling shutter [Fig. 4(d)]. A demonstration of the axial scan is shown in Fig. 5, in which each camera frame corresponds to a particular lens power in a series of linearly increasing lens powers.

**Fig. 6:**
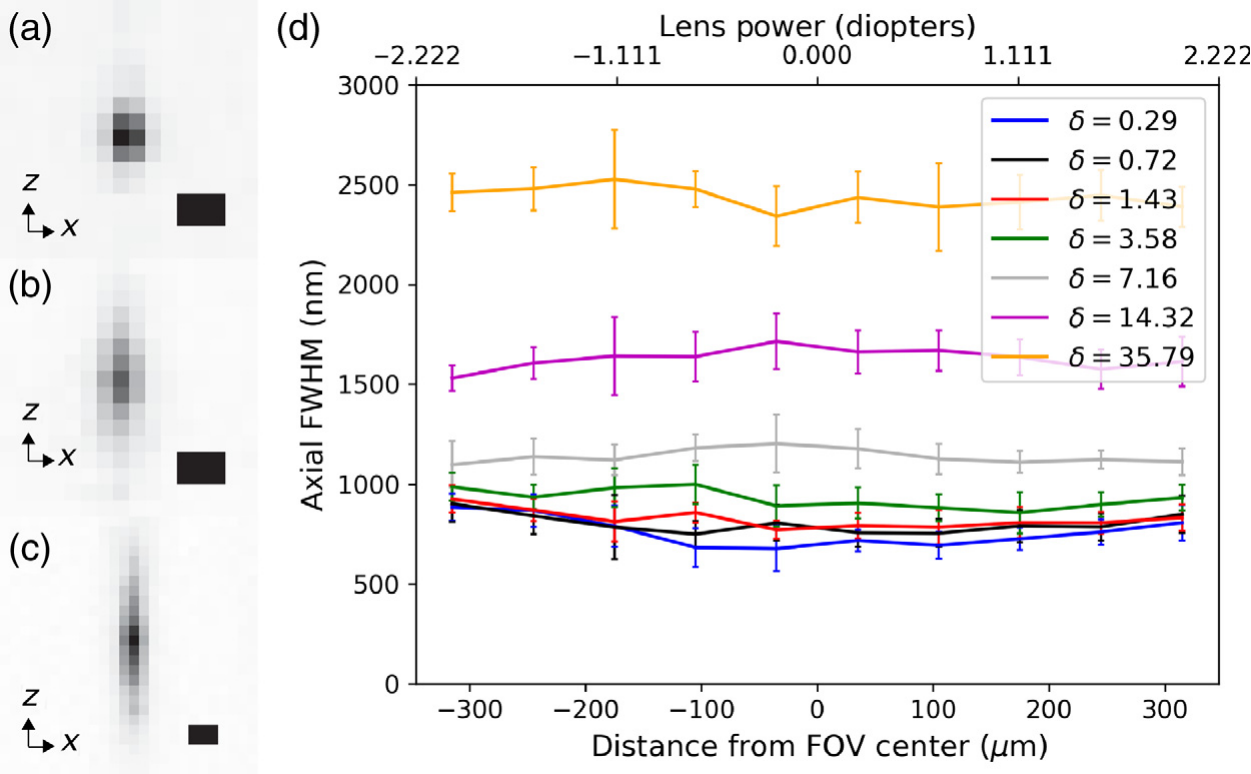
Characterization of the axial FWHM. (a)–(c) Inverted axial PSFs at the center of the FOV with δ, the ratio of the shutter width to the confocal range, of 1.43, 7.16, and 37.8, respectively. Scale bars are 1  μm. (d) Axial FWHM variation along the optical axis with various shutter widths and at different distances from the nominal focus at the FOV center. The beads are grouped based on location into bins, each of which spans one-tenth of the FOV.

The same agarose beam suspension is used to characterize the lateral and axial microscope resolution as a function of rolling shutter width. We define the parameter δ as the ratio of the shutter width (at the sample plane) to the 2.3-μm beam confocal range. Three-dimensional images are captured by stage scanning the bead suspension through the synchronized beam scan, which acquires the 3D point spread function (PSF). The lateral full-width at half maximum (FWHM), which is independent of slit width, is 504±17  nm across the FOV. The axial resolution and its dependency on slit width and location are shown in Fig. 6. At the center of the FOV with a 13-μm (two pixel) shutter width, the FWHM is 720±55  nm, and it gradually increases to 885±65  nm and 807±90  nm at the left and right edges, respectively. The deterioration of the resolution was expected; the maximum PA NA required to shift the beam focus from the center to the edge is 0.010, while 0.008 is the maximum value for diffraction-limited performance.

**Fig. 7:**
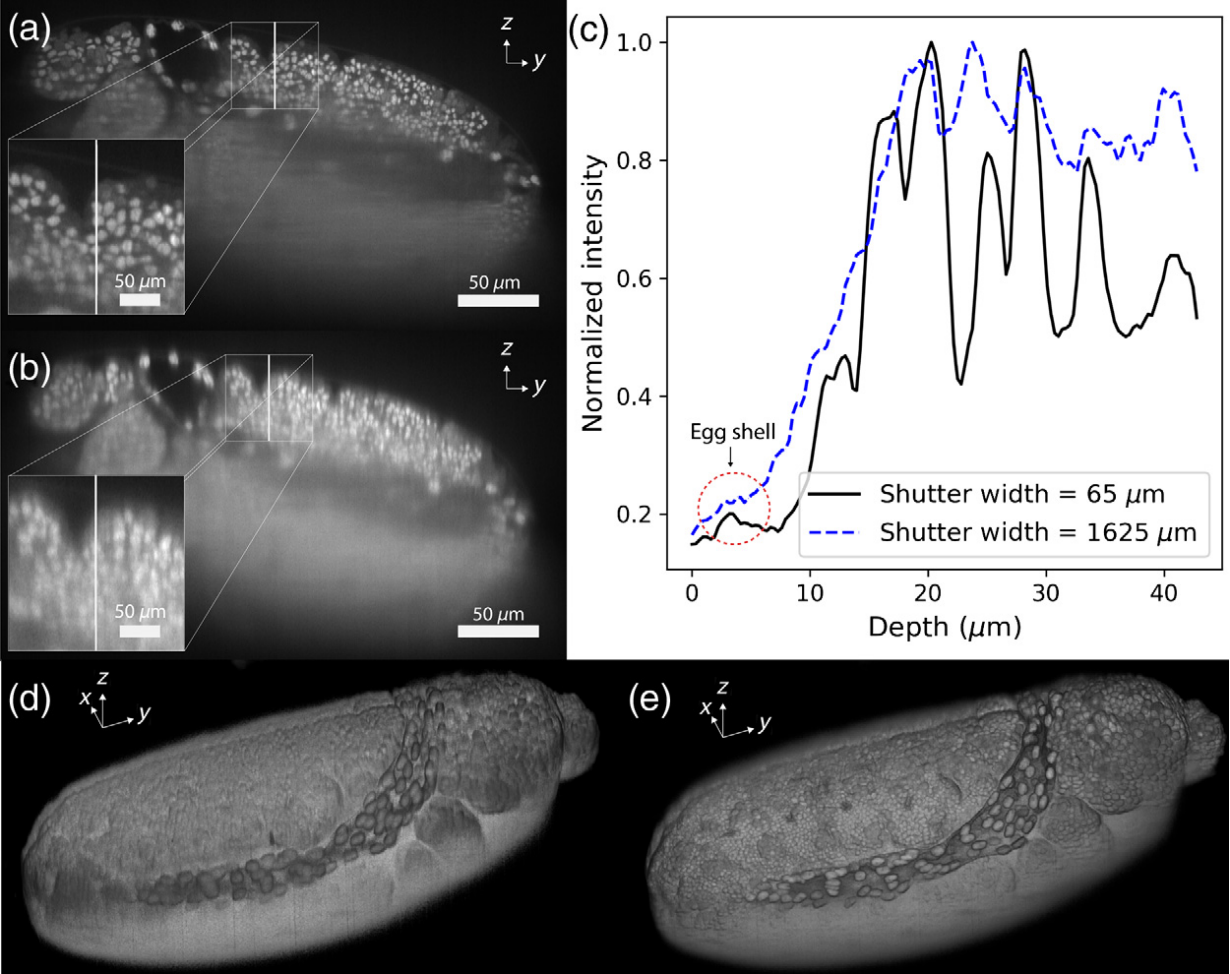
Comparison of images of an 8-h-old GFP-labeled *Drosophila* embryo captured at low and high axial resolutions. (a) and (b) YZ section from a 3D *Drosophila* image using a rolling shutter width of 65 and 1625  μm, respectively. The white line indicates the line over which the profile in (c) is taken. (c) Normalized intensity profile from the surface to the 45  μm of the sample. (d) and (e) The surface of the reconstructed 3D volumes with rolling shutter widths of 65 and 1625  μm, respectively.

We demonstrated our technique on an approximately 8-h-old *Drosophila* embryo from a line of *Drosophila* that expresses nuclear green fluorescent protein (GFP) [Fig. 7]. Embryos were collected from an apple juice plate after 8 h, dechorionated, and then suspended in glass capillaries using a 1% agarose gel. The rubber plunger of the capillary is used to push the suspension just outside the glass, so the light sheet passes through the sample without interacting with the glass. A brief calibration of the light sheet axial scan was performed once the *Drosophila* embryo was added to the system.

Based on our system characterization, we chose a rolling shutter width of 65  μm, which sacrifices a small amount of resolution in the FOV center for five times more signal compared with the two-pixel shutter. To demonstrate the increased resolution, we compared 3D images acquired using a 65-μm shutter with that of the 1625-μm shutter case, which results in an FWHM similar to conventional LSFM systems using a static light sheet [Figs. 7(a) and 7(b)]. The line profile through the first 45  μm of the sample along z clearly shows the resolution increase [Fig. 7(c)]; deeper imaging was limited by scattering in the specimen and could benefit from multiple views. With the increased axial resolution, it is possible to distinguish the thin egg shell of the sample in the proximity of other structures. The same specimen is shown in 3D [Figs. 7(d) and 7(e)], which enables visualization of the Drosophila embryo surface in great detail.

To demonstrate this technique at increased frame rates, we reduced the camera area of interest and acquired a rapid 3D image sequence for a 14-h-old GFP-labeled *Drosophila* embryo.^8^ The acquisition runs at 140 frames per second using a 688×1400  px area of interest, with each pixel exposure lasting 96  μs, which was the highest frame rate that we used. The short exposure time combined with the low power to reduce photobleaching resulted in a moderately noisy 3D acquisition, which highlights the inherent trade-offs associated with this technique. The short pixel exposure means that relatively high powers often need to be used to achieve adequate signal-to-noise ratio (SNR) levels in these conditions.

## 5 Conclusion

**Fig. 8:**
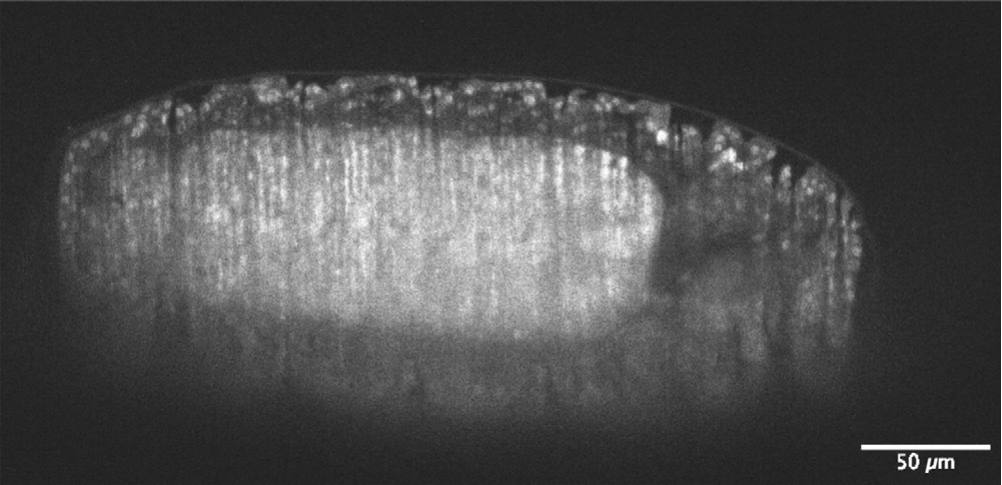
3D acquisition of a nuclear GFP-labeled *Drosophila* embryo at 14-h-old. The imaging rate is 140 frames per second (Video 2, MOV, 6 MB [URL: https://doi.org/10.1117/1.JBO.25.10.106504.2]).

Sub-micron isotropic resolution has been demonstrated using a PA to perform an axial scan in synchronization with the rolling shutter of a modern sCMOS camera. Our technique achieves sub-micron isotropic resolution in real time and utilizes a fixed readout direction without compromising on speed for cameras with readout rates as fast as 2.85  μs. The scanning is straightforward to calibrate and lends itself to automation. A blaze phase can be added to the cylindrical phase to axially scan at different planes normal to the detection axis, which can be useful for autofocus. One drawback with our approach is the efficiency of the PA, which is 50% in the worst case (Fig. 8).

The presented system can be readily adapted for use with lower equal magnification objectives, such as 5× or 10×, for large area volumetric imaging, which may be of interest for cleared tissue imaging. Use of high magnification objectives requires a PA with a smaller element size as scanning to the edge of the FOV in that case will require a larger NA than currently supported. With high magnifications, the FOV decreases (proportional to the focal length of the objective, $f_{\mathrm{obj}}$), restricting how far the beam must be scanned. However, the PA lens power required to scan to a given location from the FOV center is proportional to $\frac{1}{f_{\mathrm{obj}}^{2}}$ [Eq. (3)], leading to an overall scaling factor for the maximum PA lens power to scan to the FOV edge as magnification changes of $\frac{1}{f_{\mathrm{obj}}}\propto M_{\mathrm{obj}}$. Additionally, as Chakraborty et al.^22^ point out, as the NA of the objectives becomes large (≥0.4), it becomes increasing important to use a matched objective in the illumination path to correct for aberrations along the axial scan path. In principle, with a well-characterized IO and smaller element size, a PA could offer an alternative correction scheme for this response. Simply adding the spatially dependent compensating phase to the cylindrical phase along the length of the scan would restore the beam quality.

This light sheet microscope is best suited for specimens with robust fluorophores or those requiring a single 3D scan, such as cleared tissue. In these applications, the effects of photobleaching can be neglected and high laser powers can be used to achieve adequate SNR while continuing to operate with high axial resolution at maximal frame rates. Faster sCMOS cameras have recently become available from Hamamatsu with row readout rates as low as 4.9  μs. Even with this camera, which operates twice as fast as the one used in our demonstration, our PA device would still operate fast enough for a camera-limited performance of up to 100 full frames per second.
